# Application of tongue image characteristics and oral-gut microbiota in predicting pre-diabetes and type 2 diabetes with machine learning

**DOI:** 10.3389/fcimb.2024.1477638

**Published:** 2024-11-04

**Authors:** Jialin Deng, Shixuan Dai, Shi Liu, Liping Tu, Ji Cui, Xiaojuan Hu, Xipeng Qiu, Tao Jiang, Jiatuo Xu

**Affiliations:** ^1^ Department of College of Traditional Chinese Medicine, Shanghai University of Traditional Chinese Medicine, Shanghai, China; ^2^ School of Computer Science, Fudan University, Shanghai, China

**Keywords:** tongue diagnosis, oral-gut microbiome, prediabetes mellitus, type 2 diabetes mellitus, diagnostic model

## Abstract

**Background:**

This study aimed to characterize the oral and gut microbiota in prediabetes mellitus (Pre-DM) and type 2 diabetes mellitus (T2DM) patients while exploring the association between tongue manifestations and the oral-gut microbiota axis in diabetes progression.

**Methods:**

Participants included 30 Pre-DM patients, 37 individuals with T2DM, and 28 healthy controls. Tongue images and oral/fecal samples were analyzed using image processing and 16S rRNA sequencing. Machine learning techniques, including support vector machine (SVM), random forest, gradient boosting, adaptive boosting, and K-nearest neighbors, were applied to integrate tongue image data with microbiota profiles to construct predictive models for Pre-DM and T2DM classification.

**Results:**

Significant shifts in tongue characteristics were identified during the progression from Pre-DM to T2DM. Elevated Firmicutes levels along the oral-gut axis were associated with white greasy fur, indicative of underlying metabolic changes. An SVM-based predictive model demonstrated an accuracy of 78.9%, with an AUC of 86.9%. Notably, tongue image parameters (TB-a, perALL) and specific microbiota (*Escherichia*, *Porphyromonas-A*) emerged as prominent diagnostic markers for Pre-DM and T2DM.

**Conclusion:**

The integration of tongue diagnosis with microbiome analysis reveals distinct tongue features and microbial markers. This approach significantly improves the diagnostic capability for Pre-DM and T2DM.

## 1 Introduction

Diabetes mellitus (DM) is a multifactorial endocrine and metabolic disorder triggered by a combination of genetic predisposition and environmental influences, which disrupt insulin secretion and impair insulin sensitivity, ultimately resulting in multi-organ dysfunction and potential organ failure (Standl et al., 2019). In China, the prevalence of type 2 diabetes (T2DM) among adults is approximately 10.9%, with pre-diabetes (Pre-DM) affecting around 35.7% of the population (Wang et al., 2017). T2DM, a polygenic condition, arises from a combination of hereditary and environmental factors, with insulin resistance (IR) and beta-cell dysfunction (reduced insulin production) as its hallmark features (Paquette et al., 2023). Pre-DM, defined by impaired fasting glucose (FBG) and/or impaired glucose tolerance, presents a significant risk, as up to 21% of individuals progress to T2DM within three years (Eades et al., 2014). Thus, early intervention during the pre-diabetic phase is one of the most effective strategies for mitigating the onset of T2DM.However, the indicators and underlying mechanisms predisposing to the conversion of Pre-DM individuals to T2DM remain unclear (Takeuchi et al., 2023).

Tongue diagnosis plays a fundamental role in traditional Chinese medicine (TCM) diagnosis, characterized by features such as tongue shape, color, texture, back, coating color, and thickness. TCM relies on these visual characteristics to infer disease progression and type. However, traditional visual observation in TCM lacks the ability to objectively quantify these features (Thirunavukkarasu et al., 2024). Utilizing computer image processing enables the segmentation of various tongue regions, the automatic extraction of spectral parameters, and the identification of distinct features, including region division, color, texture, and shape. These metrics offer a more precise description of the tongue’s properties. In addition, deep learning approaches provide a more comprehensive analysis of tongue images, allowing for an enhanced understanding of the underlying pathology. In previous research, we developed a robust classification system specifically for diabetic tongues (Li et al., 2022), leveraging a deep learning model to evaluate a substantial number of tongue images and establish optical characteristics associated with diabetes (Jiang et al., 2021). Building on these results, a method was devised to correlate tongue image data with diabetes, supporting the potential of tongue features as early biomarkers for diagnosing prediabetes and diabetes (Li et al., 2021a). The tongue, being the initial segment of the digestive tract, is also intimately linked to the oral microbiome, which plays a crucial role in maintaining oral ecological balance and is associated with the onset and progression of systemic diseases (Gao et al., 2018). Recent studies suggest a significant interplay between oral and intestinal microbiota, with disruptions in intestinal microbiota implicated as a risk factor for chronic conditions such as T2DM, gastrointestinal cancers, and neurological disorders (Tuganbaev et al., 2022; Pan et al., 2024).

In light of the significant involvement of tongue-coated microbiota (oral microbiota) in TCM tongue diagnosis, this study aimed to explore the relationship between alterations in TCM tongue patterns and the oral-gut microbiota axis during diabetes progression. The objective was to offer early risk indicators for T2DM, enabling timely interventions, while simultaneously advancing the scientific comprehension of tongue diagnosis within the framework of TCM.

## 2 Methods

### 2.1 Study subject recruitment

The study encompassed individuals undergoing physical examinations at the Shanghai Gaohang Community Service Center between 2022 and 2023. Following the application of inclusion and exclusion criteria, 28 healthy controls, 30 individuals with Pre-DM, and 37 T2DM patients were selected. Informed consent, approved by the ethics committee of Shuguang Hospital, affiliated with Shanghai University of TCM, was obtained from all participants.

Participants were considered eligible for the Pre-DM group based on one or more of the following criteria: (1) FBG ranging from 5.6 to 6.9 mmol/L; (2) 2-hour postprandial blood glucose (2hPG) between 7.8 and 11.0 mmol/L; (3) Glycosylated hemoglobin (HbA1C) levels between 5.7% and 6.4%. For T2DM, inclusion required meeting one or more of the following: (1) random blood glucose ≥11.1 mmol/L; (2) FBG ≥7.0 mmol/L; (3) 2hPG ≥11.1 mmol/L; (4) HbA1C ≥6.5%.

The exclusion criteria included: (1) patients diagnosed with type 1 or specific types of DM, or those experiencing acute complications such as ketoacidosis; (2) recent use of antibiotics, probiotics, traditional Chinese medicines, or immunosuppressive drugs within the past two weeks; (3) coexisting oral conditions or complications, including periodontitis, pulpitis, or oral cancer; (4) individuals with severe systemic diseases, such as malignant tumors, immune disorders, or hematological diseases; (5) pregnant or breastfeeding women; (6) individuals unable to provide fully informed consent due to mental health symptoms, behavioral disorders, or cognitive impairments; (7) participants with distinct dietary habits; (8) participants with irregular bowel movements; (9) smokers.

### 2.2 Data and sample collection

The clinical characteristics of the enrolled patients were examined in this cross-sectional study, with data collected on demographic factors such as age, gender, body mass index (BMI), and waist-to-hip ratio (WHR). Personal lifestyle factors included smoking and drinking history, along with dietary preferences categorized as spicy, sweet, or no specific preference. The laboratory-developed TCM Clinical Diagnosis Record Form documented key symptoms, including dry mouth, bitter taste, constipation, and diarrhea. Blood pressure was assessed according to World Health Organization (WHO) criteria, classifying hypertension into grade 1 (140–159 mmHg systolic/90–99 mmHg diastolic), grade 2 (160–179 mmHg systolic/100–109 mmHg diastolic), and grade 3 (systolic ≥ 180 mmHg/diastolic ≥ 110 mmHg).

**Figure 1:**
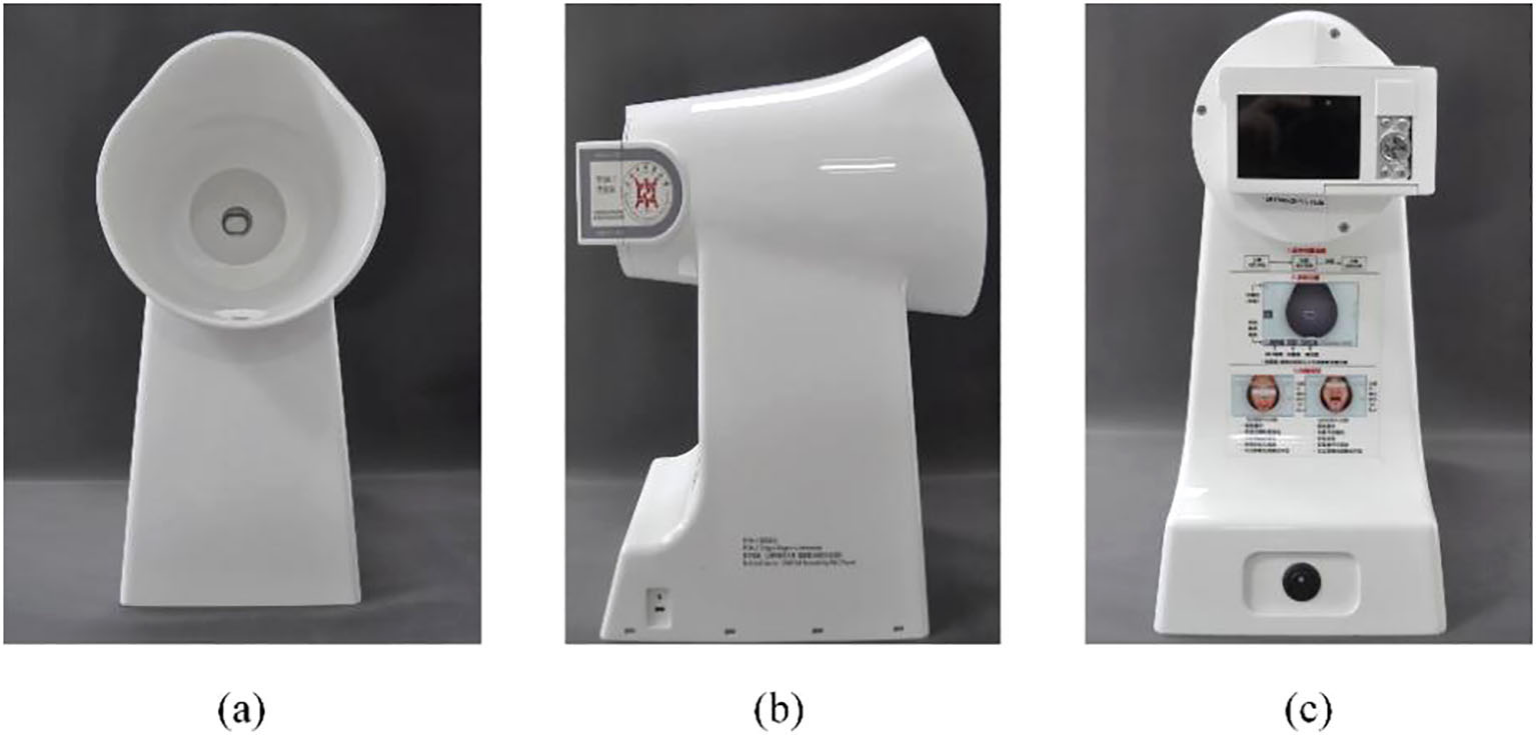
Display of TFDA-1 Tongue Diagnostic Instrument. **(A)** was the front picture, **(B)** was the side picture, and **(C)** was the shooting interface picture.

The tongue image acquisition process utilized the Tongue Diagnostic Instrument (TFDA-1), a device engineered by the Intelligent Diagnostic Laboratory at Shanghai University of Traditional Chinese Medicine, as depicted in **Figure 1**. Detailed data collection procedures can be found in our earlier publication (Soper, 2021). Key technical specifications of the device included a manual operating mode, 1/125 shutter speed, F6.3 aperture setting, ISO sensitivity of 200, correlated color temperature ranging from 4500K to 7000K, and illumination at 4800 ± 10% (unit: lx).

Oral microbiota samples were obtained from the central region of the tongue dorsum using aseptic pharyngeal swabs, with at least ten rotational movements. Stool samples were self-collected by participants in the morning using a sterile fecal sampler, targeting the central portion of the stool. Following cryopreservation, the samples were immediately dispatched to researchers on the same day. Each specimen was sealed in sterile, enzyme-free Eppendorf tubes, kept on ice, and transferred to a −80°C freezer within 30 minutes for preservation prior to sequencing. Participants were instructed to fast before sample collection.

Tongue image features were extracted using the established methods previously developed by our research group. An intelligent quality assessment model was employed to screen all collected tongue images, ensuring they met the required quality standards (Jiang et al., 2021). Feature extraction was performed using the adversarial generation network, Tongue-GAN, as described in our earlier publications (Li et al., 2021b; Jiang et al., 2022).

### 2.3 DNA extraction and 16S full-length library construction

Bacterial DNA from the tongue dorsum and fecal samples was extracted using a swab genomic DNA extraction kit (CW2654, CwBiotech, Beijing, China) and an intestinal DNA extraction kit (TIANamp Stool DNA Kit, DP328, Tiangen Biotech, Beijing, China), respectively. The 16S rDNA full-length assembly sequencing technology (16S-FAST) enabled species-level classification through analysis of bacterial ribosomal 16S RNA sequences, encompassing nine variable and ten conserved regions (Karst et al., 2018). For both qualitative and quantitative assessment, as well as quality control, 10 ng of DNA was utilized. Splice and link libraries were constructed, followed by data assembly from electrophoresis and Qubit concentration measurements to ensure quality before proceeding with sequencing on the Illumina NovaSeq 6000 platform (Illumina, USA). The methodology has been thoroughly detailed in a previous study by members of the research team (Guo et al., 2023).

### 2.4 Bioinformatic analysis

A cloud platform (https://www.genescloud.cn/home) was employed for sequence analyses, utilizing QIIME2 (2019.4), R language (v3.2.0), the ggplot2 package, and Python. ASV-level alpha diversity, including Shannon diversity indices, was calculated via the ASV table in QIIME2 and visualized through box plots. To assess the significance of differences, the Kruskal-Wallis rank sum test followed by Dunn’s *post-hoc* test was applied. Ranked abundance curves at the ASV level were generated to evaluate richness and evenness across samples. Beta diversity analysis, leveraging UniFrac distance metrics, explored microbial community structural variation, visualized through principal coordinate analysis (PCoA) and hierarchical clustering. Microbial structure differentiation among groups was quantified using permutational multivariate analysis of variance (PERMANOVA) in QIIME2. Linear discriminant analysis effect size (LEfSe) analysis identified differentially abundant taxa among groups under default settings. Random forest analysis in QIIME2, with default parameters, was employed to classify samples from distinct groups, utilizing nested stratified k-fold cross-validation for automated hyperparameter optimization and sample prediction. Co-occurrence network analysis was conducted via SparCC, with a pseudo-count set to 106. Correlation coefficient cutoffs were established at 0.70 using random matrix theory-based methods in the R package RMThreshold, with Cytoscape (v3.9.0) constructing the network visualization. The R language facilitated analysis of the network’s topological structure, with key species identified through topological indices and visualized using the ZiPi plot. Phylogenetic Investigation of Communities by Reconstruction of Unobserved States (PICRUSt2) predicted microbial functions based on MetaCyc (https://metacyc.org/).

### 2.5 Machine learning methods

Logistic regression with backward selection was applied, incorporating L2 regularization, a tolerance of 1e-4, an inverse regularization strength (C) of 1.0, and the lbfgs solver, with a maximum of 100 iterations. Tongue image features, clinical indicators, and microbial data were screened for Pre-DM and T2DM classification, allowing the removal of insignificant variables while addressing multicollinearity (Chicco et al., 2021). Model fit was evaluated via maximum likelihood and the Hosmer-Lemeshow test. A combined tongue-microbiota classification model for Pre-DM and T2DM was subsequently developed and validated. To ensure robustness, 5-fold cross-validation was employed, partitioning data into five subsets and averaging performance metrics such as ROC AUC, accuracy, sensitivity, and specificity (Lin et al., 2020; Soper, 2021). Python 3.10.9 facilitated machine learning techniques aimed at capturing non-linear relationships, utilizing models like support vector machines (SVM), random forests (RF), gradient boosting, adaptive boosting (AdaBoost), and K-nearest neighbors (KNN). Classification results were calculated using the sklearn library (Version 1.3.1).

### 2.6 Statistical analysis

Data analysis utilized SPSS v. 25.0 (IBM Corp., Armonk, NY, USA). Variable distribution normality and variance homogeneity were assessed using the Shapiro–Wilk and Levene tests, respectively. For data meeting normal distribution and variance homogeneity criteria, a t-test was applied; otherwise, non-parametric methods were employed. Categorical variables were analyzed using Fisher’s exact test, while continuous variables were evaluated via the Wilcoxon rank-sum test. Relationships among independent variables were examined through Spearman’s rank correlation, with p-values adjusted for multiple comparisons using the Bonferroni correction.

## 3 Results

### 3.1 Baseline clinical characteristics of the study cohort

**Figure 2:**
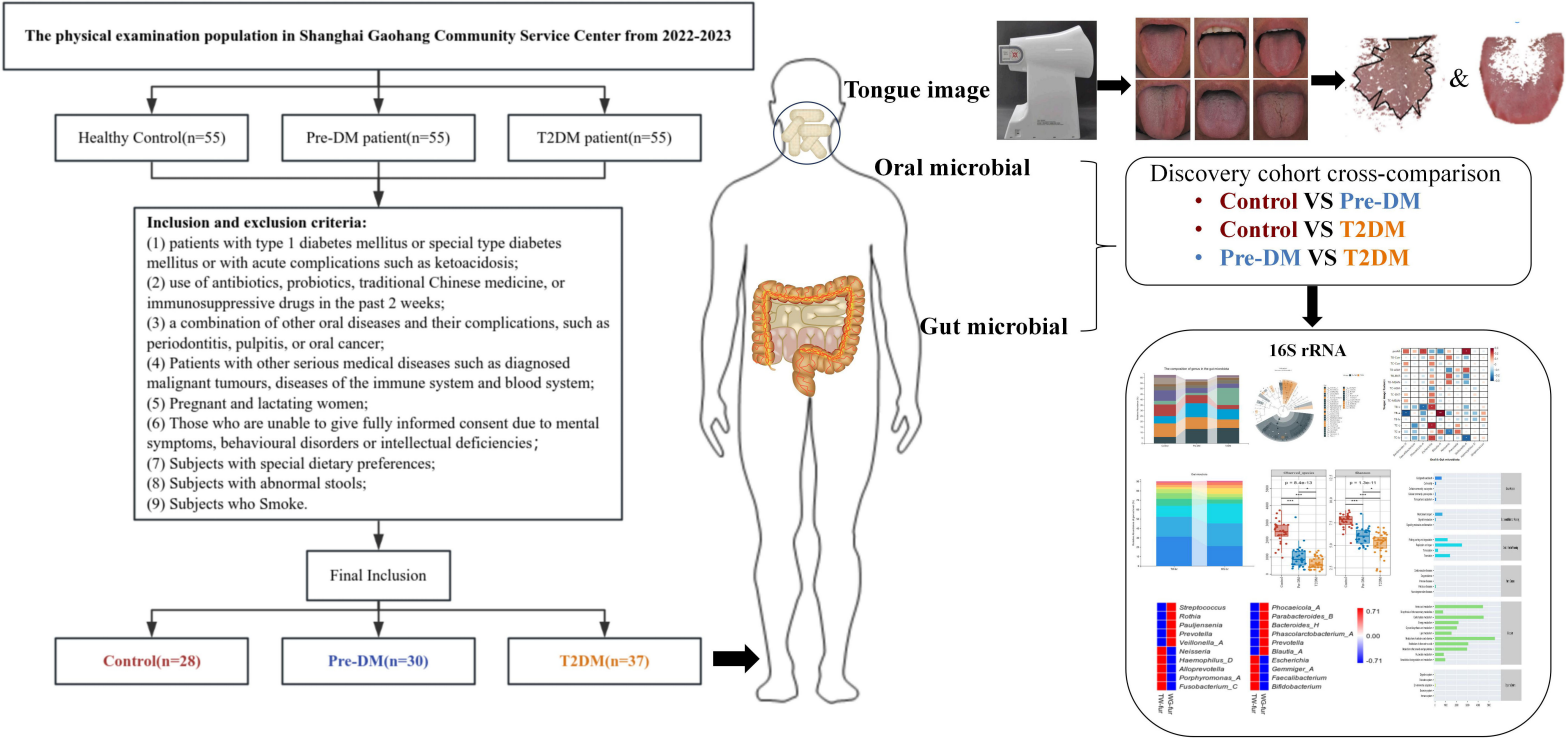
Flow Chart of the Clinical Cohort. A total of 28 Control patients, 30 Pre-DM patients, and 37 T2DM patients were selected from the screened population. Tongue images were captured via TFDA-1, followed by analysis of tongue image characteristics. Additionally, oral and fecal samples were obtained for bioinformatic profiling, utilizing 16S-FAST for subsequent analysis.

Following the screening process, 28 healthy controls, 30 Pre-DM patients, and 37 T2DM patients were included in the study (**Figure 2**), with baseline clinical characteristics detailed in **Table 1**. The Pre-DM and T2DM groups exhibited older average ages compared to the control group, although no significant age difference was observed between the Pre-DM and T2DM groups. Hypertension was identified as a risk factor for diabetes, with a markedly higher prevalence of hypertension among T2DM patients. BMI and WHR measurements indicated greater obesity in both the Pre-DM and T2DM groups relative to the control group.

**Table 1:** Characteristics of the Discovery Cohort.

		Control (n=28)	Pre-DM (n=30)	T2DM (37)	*P*
Male Sex (%)		8 (28%)	7 (23%)	15 (40%)	0.296
Age (Mean ± SD)		61.96 ± 6.19	68.13 ± 9.31^**^	69.70 ± 6.59^**^	0.000
BMI (kg/m^2^)		24.13 ± 2.85	24.65 ± 4.02	24.72 ± 2.63	0.684
WHR (Mean ± SD)		0.89 ± 0.05	0.90 ± 0.06	0.91 ± 0.07	0.480
BP (%)	None	28 (100%)	19 (63%)	11 (29%)	0.000
	Primary	0	8 (27%)	12 (32%)	
	Secondary	0	3 (10%)	11 (29%)	
	Tertiary	0	0	3 (10%)	
FBG (mmo/L)		5.55 ± 0.37	5.56 ± 0.59	7.77 ± 3.07**^##^	0.000
2hPG (mmo/L)		\	8.25 ± 1.99	10.99 ± 3.73^#^	0.002
HbA1C (%)		\	5.96 ± 0.37	7.56 ± 2.88^#^	0.001

** Compared with Control group, *P* < 0.01; #Compared with Pre-DM group, *P* < 0.05; ##Compared with Pre-DM group, *P* < 0.01.

### 3.2 The change of tongue

**Figure 3:**
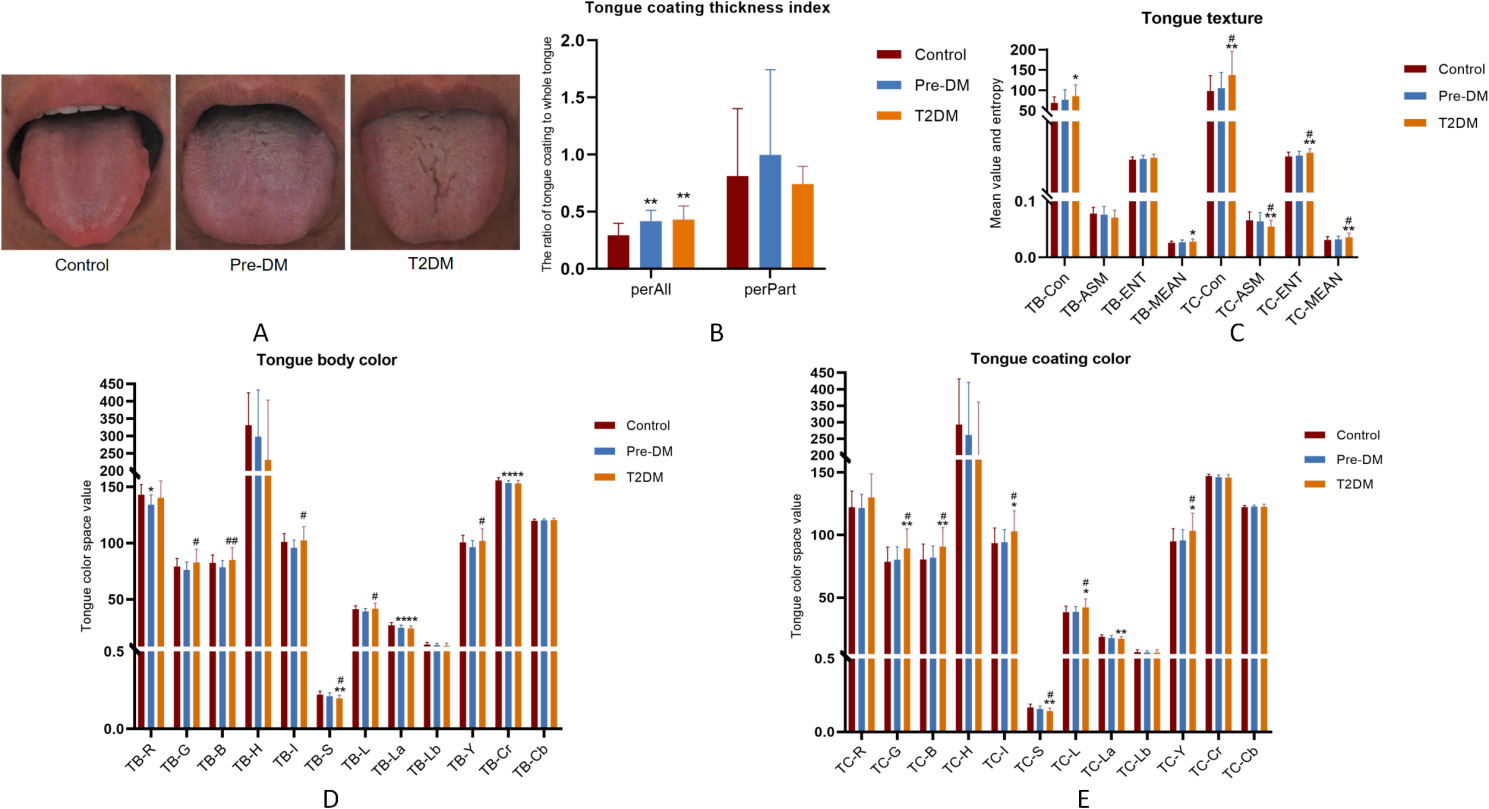
Tongue image features of participants. **(A)** Three groups of tongue images;**(B)** tongue coating thickness index; **(C)** tongue texture; **(D)** tongue body color; **(E)** tongue coating color; *Compared with Control group, *P* < 0.05; ** Compared with Control group, *P* < 0.01; #Compared with Pre-DM group, *P* < 0.05; ##Compared with Pre-DM group, *P* < 0.01.

Following the extraction of tongue image features, three sets of crowd computer tongue image parameters were identified. Notably, a significant increase in preALL was observed in both the Pre-DM and T2DM groups, indicating a marked thickening and greasiness of the tongue coating. The Con* and MEAN* values for both the tongue body and coating in the T2DM group were substantially higher compared to the other groups. Furthermore, the ASM* and ENT* values for the tongue coating displayed significant variation when compared to the Control and Pre-DM groups. These results suggest a progressive transition in the tongue texture from smooth to rough, indicative of aging as diabetes progresses. Analyzing the color parameters of the tongue and coating, a gradual shift towards paler and whiter shades was noted, as shown in **Table 2** and **Figure 3**.

**Table 2:** Tongue image features of participants.

	Control(n=28)	Pre-DM(n=30)	T2DM(37)	*P*
perAll	0.294 ± 0.106	0.418 ± 0.095**	0.431 ± 0.120**	0.000
perPart	0.812 ± 0.591	0.997 ± 0.745	0.742 ± 0.156	0.067
TB-Con	68.971 ± 15.086	76.913 ± 24.674	85.539 ± 27.800*	0.011
TC-Con	98.086 ± 38.085	105.247 ± 38.683	137.266 ± 58.421**#	0.002
TB-ASM	0.078 ± 0.011	0.076 ± 0.015	0.071 ± 0.013	0.118
TB-ENT	1.197 ± 0.056	1.210 ± 0.080	1.237 ± 0.073	0.072
TB-MEAN	0.026 ± 0.003	0.027 ± 0.004	0.028 ± 0.005*	0.033
TC-ASM	0.066 ± 0.015	0.064 ± 0.016	0.055 ± 0.011**#	0.004
TC-ENT	1.263 ± 0.093	1.280 ± 0.093	1.342 ± 0.085**#	0.001
TC-MEAN	0.031 ± 0.006	0.032 ± 0.006	0.036 ± 0.007**#	0.003
TB-R	143.036 ± 9.187	134.167 ± 8.710*	140.324 ± 14.996	0.001
TB-G	79.214 ± 7.345	76.167 ± 6.978	82.784 ± 11.804#	0.020
TB-B	82.429 ± 7.042	78.433 ± 5.998	85.135 ± 10.942##	0.005
TC-R	122.179 ± 12.919	121.533 ± 10.894	130.135 ± 18.768	0.062
TC-G	78.607 ± 11.707	80.133 ± 10.507	89.270 ± 15.816**#	0.002
TC-B	80.464 ± 12.285	81.867 ± 9.387	90.676 ± 15.288**#	0.003
TB-H	331.587 ± 92.795	298.015 ± 134.633	231.552 ± 171.991	0.119
TB-I	101.179 ± 7.339	95.967 ± 6.775	102.48 ± 12.238#	0.005
TB-S	102.48± 12.238	0.211 ± 0.022	0.197 ± 0.021**#	0.000
TC-H	293.145 ± 138.032	261.865 ± 159.085	183.245 ± 178.388	0.180
TC-I	93.429± 12.197	94.200± 10.046	94.200 ± 10.046*#	0.007
TC-S	0.167 ± 0.023	0.156 ± 0.021	0.142 ± 0.021**#	0.000
TB-L	41.345 ± 2.998	39.294 ± 2.839	41.835 ± 4.964#	0.009
TB-a	27.066 ± 2.429	24.737 ± 2.465**	24.281 ± 2.174**	0.000
TB-b	9.963 ± 1.778	9.377 ± 1.570	8.963 ± 2.215	0.117
TC-L	38.287± 4.918	38.646 ± 4.248	42.226± 6.621*#	0.006
TC-a	18.651± 1.685	17.665 ± 0.076	17.087 ± 1.855**	0.005
TC-b	6.530 ± 1.777	6.119 ± 1.545	5.989 ± 2.528	0.560
TB-Y	100.735 ± 6.389	96.530± 5.969	102.10± 10.664#	0.009
TB-Cr	155.802 ± 2.416	153.31± 2.428**	153.105 ± 2.664**	0.000
TB-Cb	119.952 ± 1.354	120.39± 1.209	120.504 ± 1.769	0.314
TC-Y	94.880± 10.307	95.621± 8.838	103.29± 14.143*#	0.006
TC-Cr	147.005 ± 1.536	146.06± 1.831	145.848 ± 2.412	0.063
TC-Cb	122.357 ± 1.318	122.62± 1.222	122.560 ± 2.038	0.722

Tongue coating index: perAl l, perPart; Texture indicators: CON (contrast degree), ASM (Angle degree second moment), ENT (entropy value), MEAN (average value); The color index comes from the RGB, HSI, Lab, YCrCb four color space, in which R (red value), G (green value), B (blue value), H (hue), S (color saturation), I (luminance), L (lightness), a (red-green axis), b (Yellow-blue axis), Y(yield of light), Cr (red signal and the brightness value of differences), Cb (the difference between the blue signal and the luminance value); *Compared with Control group, *P* < 0.05; ** Compared with Control group, *P* < 0.01; #Compared with Pre-DM group, *P* < 0.05; ##Compared with Pre-DM group, *P* < 0.01.

### 3.3 Alerted diversity of the oral and gut microbiota of participants

**Figure 4:**
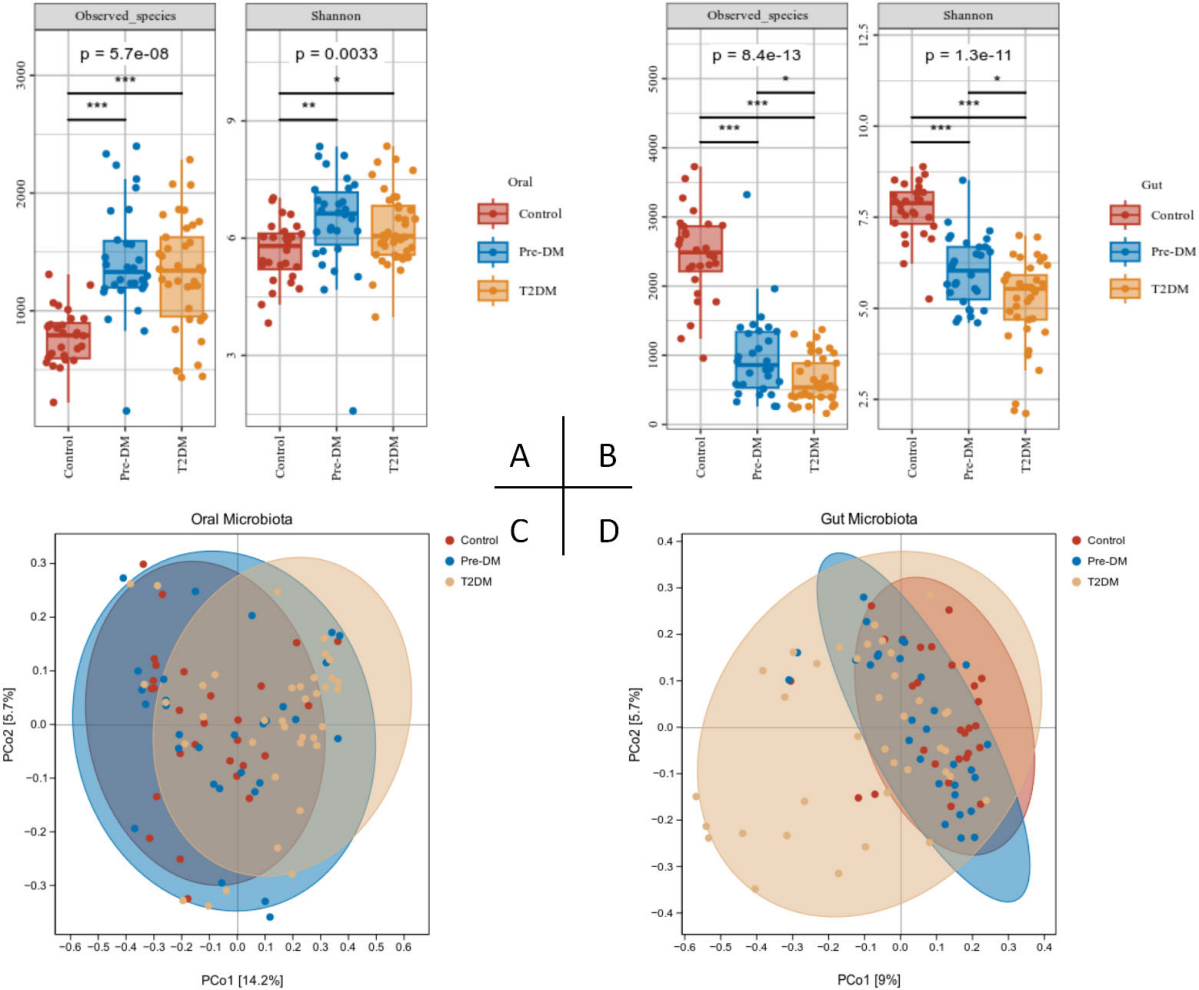
Alterations in the oral and gut microbiota diversity in Pre-DM and T2DM patients were presented as follows: **(A)** Oral microbiota α-diversity, assessed via Observed species count and Shannon index; **(B)** Gut microbiota α-diversity, similarly evaluated with Observed species and Shannon index; **(C)** PCoA analysis of the oral microbiota; **(D)** PCoA analysis of gut microbiota across participants.

α-diversity analysis revealed a marked reduction in the richness and evenness of gut microbiota as diabetes progressed, contrasted by a significant rise in oral microbiota species abundance in both the pre-DM and T2DM groups (**Figures 4A, B**). This discrepancy may be attributed to the thickened tongue coating observed in these groups. PCoA analysis further demonstrated partial overlap between the pre-DM and control groups, while the T2DM group exhibited distinct shifts in community composition, highlighting notable alterations in microbial structure (**Figures 4C, D**).

### 3.4 Changes in the composition of the oral and gut microbiota in patients with Pre-DM and T2DM

**Figure 5:**
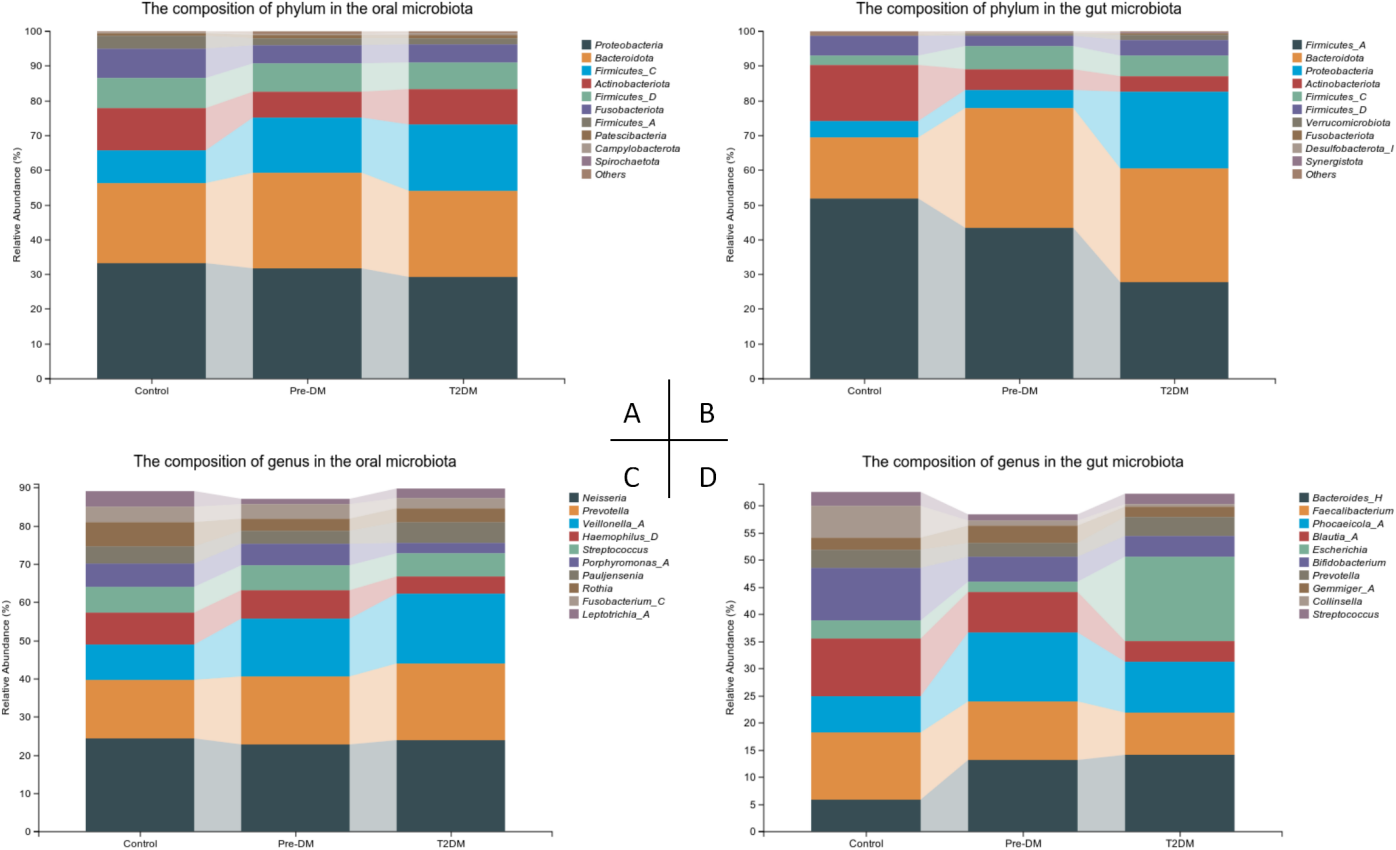
Compositional alterations of oral and gut microbiota in participants. **(A)** Stacked bar plots presenting the relative abundance of oral microbiota at the phylum level among participants; **(B)** Stacked bar plots illustrating the relative abundance of gut microbiota at the phylum level among participants; **(C)** Stacked bar plots depicting the relative abundance of oral microbiota at the genus level among participants; **(D)** Stacked bar plots demonstrating the relative abundance of gut microbiota at the genus level among participants.

Significant variations were observed in the oral and gut microbiota at both the phylum and genus levels (**Figure 5**). In the oral microbiota, diabetes progression was associated with a marked increase in *Firmicutes-C* and a decrease in *Fusobacteriota*. Notably, the Pre-DM group exhibited a rise in *Bacteroidota* and a reduction in *Actinobacteriota* compared to the control and T2DM groups. In the gut microbiota, *Bacteroidota* levels were significantly elevated, while Firmicutes-A and *Actinobacteriota* showed substantial reductions in the T2DM group relative to other groups. The microbiota shifts in Pre-DM largely mirrored those in T2DM, though the increase in *Firmicutes-C* was most prominent in the Pre-DM group. A significant rise in Proteobacteria was observed exclusively in the T2DM group.

At the genus level, *Pauljensenia* and *Veillonella-A* exhibited significant enrichment, while Neisseria showed a slight reduction in the T2DM group, aligning with the trends observed in Pre-DM. Additionally, *Haemophilus-D* and *Porphyromonas-A* were specifically reduced in the T2DM group. In the gut microbiota, Bacteroides levels increased, whereas *Faecalibacterium* and Bifidobacterium declined as the disease progressed. Notably, *Phocaeicola* exhibited the most significant increase in the Pre-DM group. *Escherichia* was markedly enriched in the T2DM group, where it accounted for the highest relative abundance.

### 3.5 Oral and gut signature microbiota in Pre-DM and T2DM

**Figure 6:**
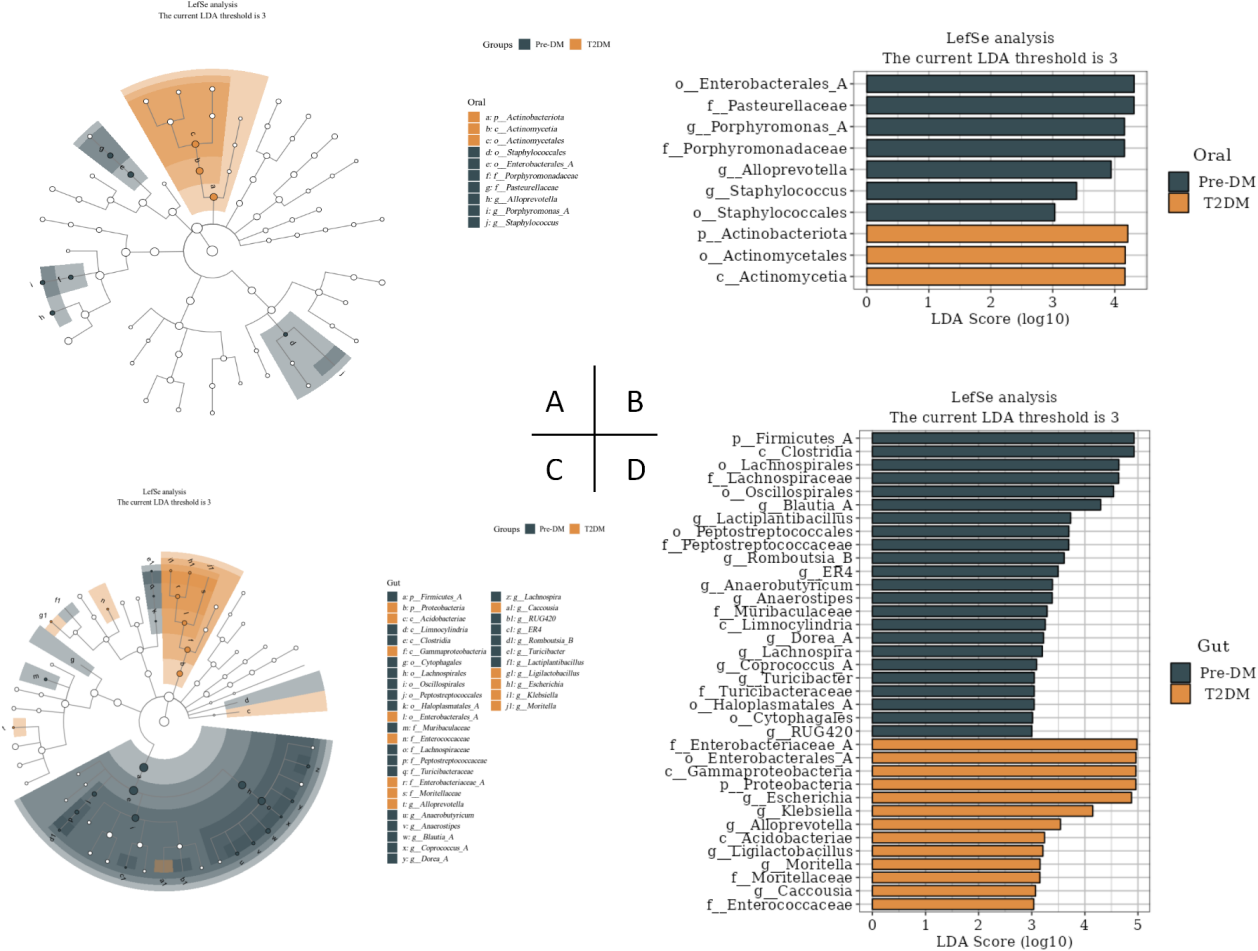
LEfSe analysis of oral and gut signature microbiota in Pre-DM and T2DM (the LDA threshold was 3). **(A)** Taxonomic branching map of oral microbiota in Pre-DM group and T2DM group; **(B)** Bar chart of oral microbiota in Pre-DM group and T2DM group; **(C)** Taxonomic branching map of gut microbiota in Pre-DM group and T2DM group; **(D)** Bar chart of gut microbiota in Pre-DM group and T2DM group.

LEfSe analysis revealed significant distinctions in tongue coating and fecal microbiota between the Pre-DM and T2DM groups (**Figure 6**). At the genus level, *Porphyromonas*, *AlloPrevotella*, and Staphylococcus within the oral microbiota, alongside *Blautia*, *Lactiplantibacillus*, and *Romboutsia-B* in the gut microbiota, emerged as potential microbial markers for Pre-DM. In contrast, *Escherichia*, *Klebsiella*, and *AlloPrevotella* in the gut microbiota were identified as potential indicators of T2DM.

### 3.6 Association analysis between tongue features and microbiota of the oral-gut axis

**Figure 7:**
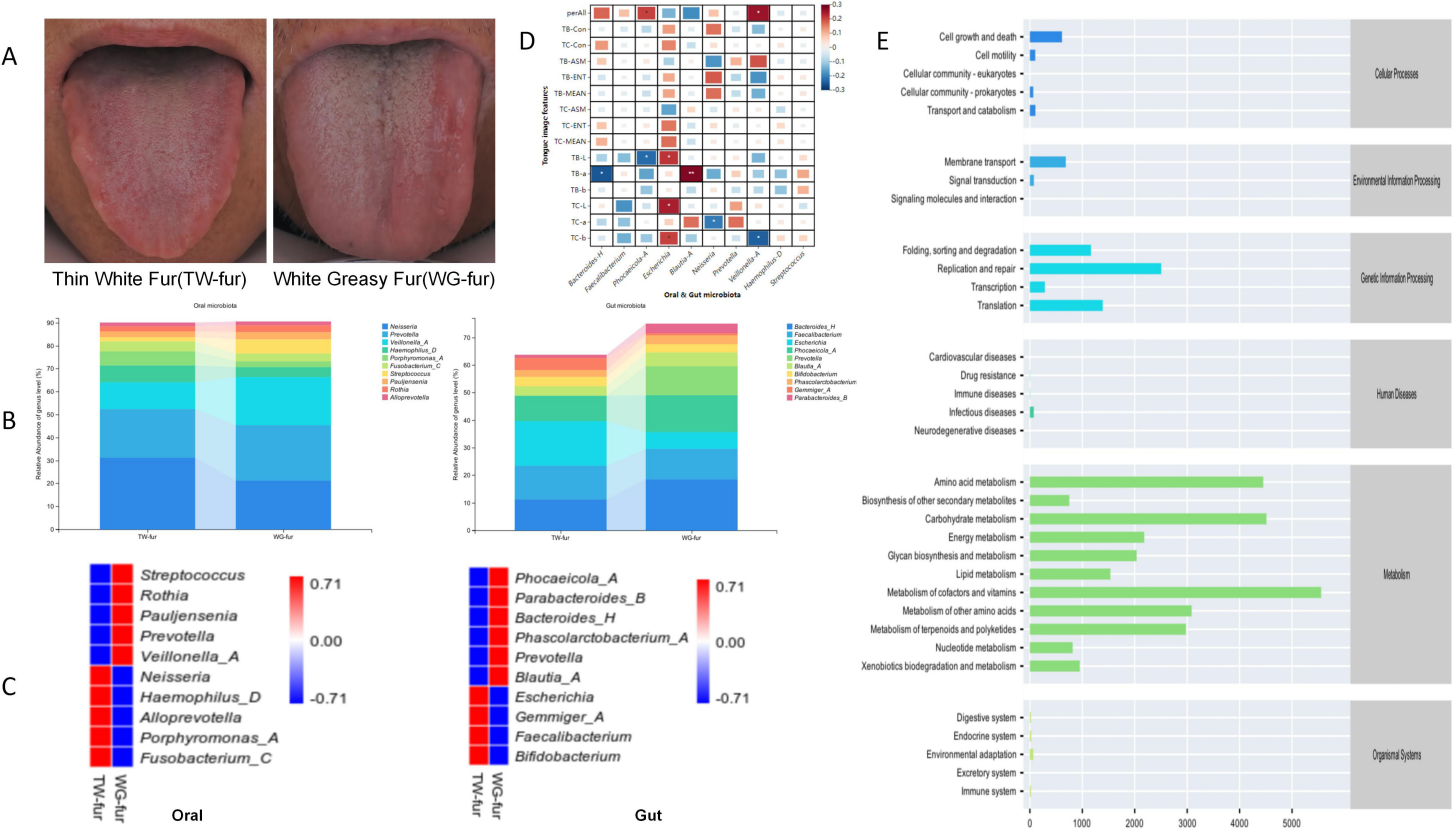
Association analysis between the microbiota of the oral-gut axis in TW-fur and WG-fur. **(A)** Two distinct groups of tongue images; **(B)** Stacked bar plots illustrating statistically significant differences in flora at the genus level; **(C)** Heat maps depicting species composition at the genus level for the two sets, with UPGMA clustering based on Pearson correlation coefficient matrix and ranked by clustering results; **(D)** Correlation heat map showing the relationship between tongue parameters and oral-gut axis microbiota (correlation coefficients as values); **(E)** Predicted metabolic pathways of tongue coating microbiota in WG-fur patients (The x-axis represents the average relative abundance of functional pathways, the y-axis lists MetaCyc functional pathways at the second classification level, and the right margin indicates the first-level pathway classification).

Abnormal FBG patients exhibiting thin white fur (TW-fur) and white greasy fur (WG-fur) were selected to investigate the relationship between tongue characteristics and the oral-gut axis microbiota. At the genus level, WG-fur was associated with elevated levels of *Veillonella-A* and Streptococcus in the oral cavity, while increased *Blautia* and *Prevotella* were observed in the gut. The majority of the altered microbiota belonged to the phylum Firmicutes. Pearson correlation analysis revealed significant associations between oral-gut microbiota and tongue parameters. Specifically, perALL showed a positive correlation with *Phocaeicola*-A and *Veillonella-A*, while TB-a exhibited the strongest correlation with *Blautia*-A (correlation coefficient 0.28, *P<*0.01). Additionally, *Escherichia* was positively correlated with multiple tongue image parameters, including TB-L, TC-L, and TC-b (**Figure 7**).

To investigate the mechanism underlying greasy fur formation, an abundance analysis of KEGG functional pathways was performed. The findings indicate that the differential alterations in tongue fur were predominantly associated with pathways related to Metabolism, with the highest abundance observed in metabolic cofactors and vitamins (**Figure 7**). According to TCM, tongue coating results from “disharmony of the viscera and fumigation of the spleen and stomach.” Increased metabolic activity elevates blood flow to the tongue, leading to a thickened coating. The metabolism of cofactors, vitamins, and other nutrients indirectly influences the tongue coating by impacting nutritional status, metabolic function, and visceral health.

### 3.7 Pre-DM and T2DM diagnostic models based on tongue image and oral and gut flora biomarker fusion

**Figure 8:**
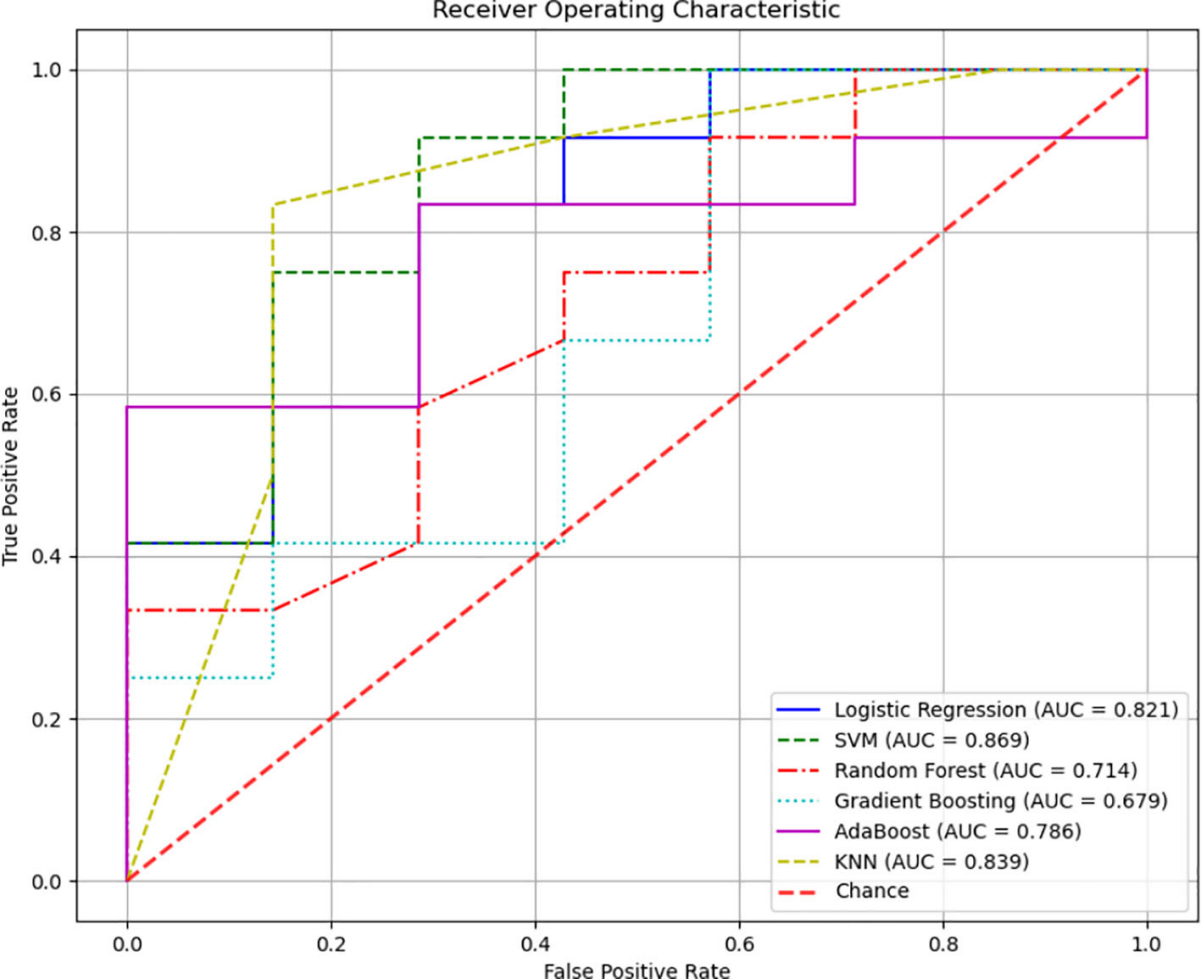
Different modeling method was adopted to establish the Pre-DM and T2DM diagnosis model of Receiver Operating Characteristic (ROC) curve.

**Figure 9:**
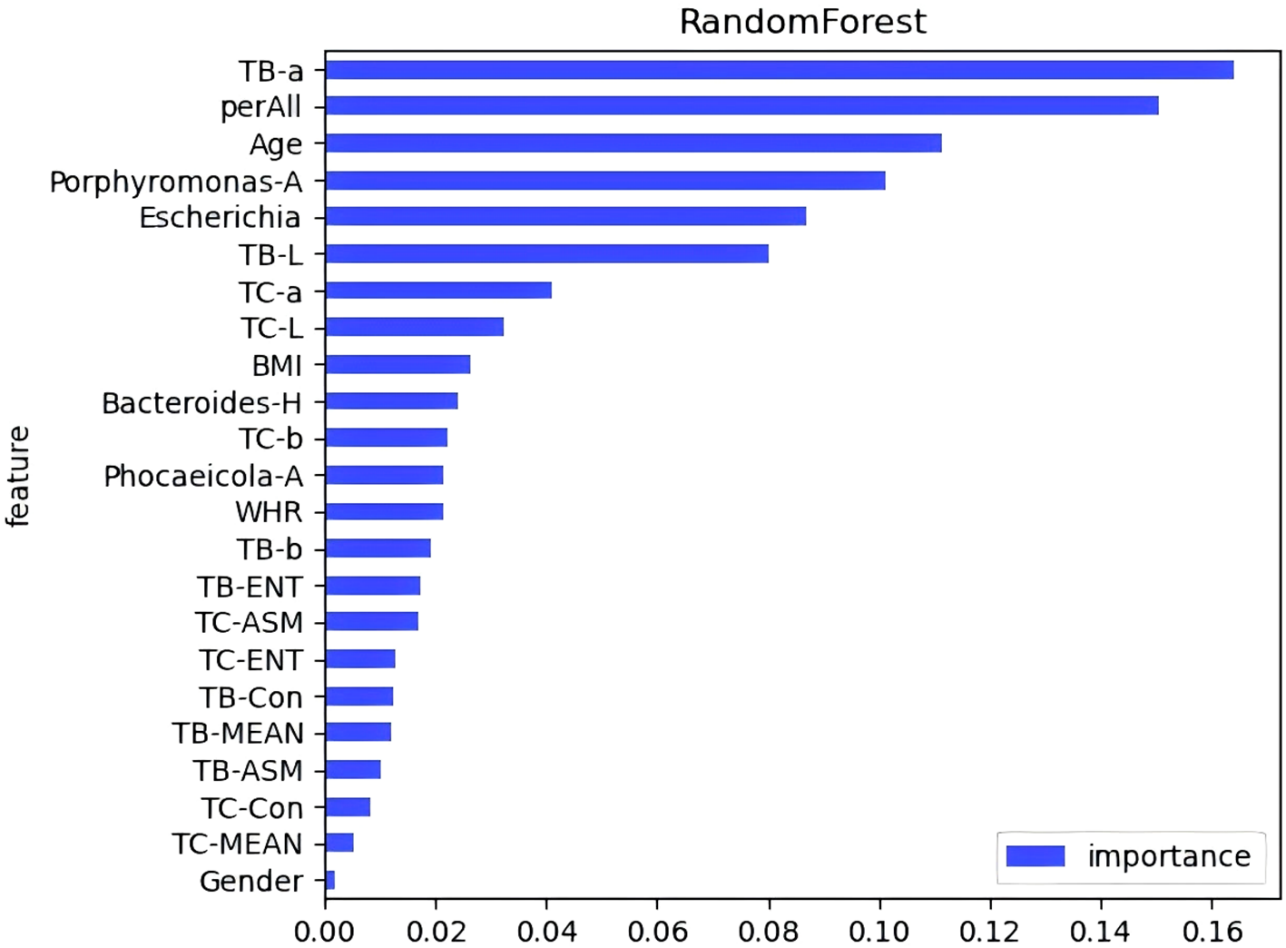
Feature importance evaluation in random forests.

A diagnostic model was developed to assess the predictive value of tongue images and microbiota in identifying Pre-DM and T2DM. Variables included gender, age, BMI, WHR, tongue image parameters (perALL, TB-Con, TC-Con, TB-ASM, TB-ENT, TB-MEAN, TC-ASM, TC-ENT, TC-MEAN, TB-L, TB-a, TB-b, TC-L, TC-a, and TC-b), and key microorganisms from the oral and gut microbiome (*Bacteroides-H*, *Phocaeicola*-*A*, *Escherichia*, and *Porphyromonas-A*). Six modelling techniques were applied—Logistic Regression, SVM, Random Forest, Gradient Boosting, AdaBoost, and KNN—to differentiate Pre-DM, T2DM, and healthy controls. Among the three classification models, SVM achieved the highest accuracy, followed by KNN, while Gradient Boosting demonstrated the poorest performance for classifying Pre-DM and T2DM (**Figure 8**, **Table 3**). Feature Importance analysis from the Random Forest model highlighted TB-a and perALL from the tongue image parameters, along with *Escherichia* and *Porphyromonas-A*, as key contributors to the model’s predictive performance, serving as primary classification indicators for Pre-DM and T2DM diagnosis (**Figure 9**).

**Table 3:** Comparison of diagnostic efficiency of six different machine learning methods.

Methods	AUC	Accuracy	Precision	Recall	F1-Score
Logistic Regression	0.821	0.737	0.733	0.917	0.815
SVM	0.869	0.789	0.750	1.000	0.857
Random Forest	0.714	0.737	0.733	0.917	0.815
Gradient Boosting	0.679	0.737	0.733	0.917	0.815
AdaBoost	0.786	0.684	0.714	0.833	0.769
KNN	0.839	0.789	0.786	0.917	0.769

AUC, Area Under the Curve.

## 4 Discussion

Tongue image diagnosis plays a central role in TCM diagnosis, with recent research indicating a strong correlation between changes in tongue appearance, coating, and the oral microbiome (Han et al., 2016; Wilbert et al., 2020; Lu et al., 2022). As the oral cavity serves as the entry point to the digestive tract, the oral and gastrointestinal microbiota—the two largest microbiomes in the human body—are intricately connected. While gut microbiota has been extensively studied, particularly its role and metabolites in the development and progression of T2DM (Li et al., 2020; Longo et al., 2023), less attention has been given to the impact of oral microorganisms on T2DM. The oral-gut microbiota axis has been identified as a key mechanism through which oral microbiota influence host diseases (Li et al., 2024b; Zhang et al., 2024b). Previous studies from our group have identified shared commensal bacteria between the tongue coating and the intestines, particularly *Prevotella* (Guo et al., 2022). However, few studies have specifically addressed the role of oral microorganisms in T2DM. This study investigates the tongue image characteristics of Pre-DM and T2DM patients, alongside the dynamic changes in the oral-gut microbiota axis, through a clinical cohort study, emphasizing the relationship between oral microbiota and tongue image characteristics at different stages of diabetes progression. We can identify diabetes-related metabolites and study the link between specific flora and systemic inflammation by analyzing the oral-gut microbiota axis.

Tongue diagnosis, rooted in the TCM theory of the visceral picture, posits that internal organs are connected to the tongue via meridians, either directly or indirectly (Zhao et al., 2013). Consequently, abnormalities in tongue coating signify systemic imbalances, including alterations in the tongue-coating microbiome (Jiang et al., 2012). The physiological state and pathological conditions of internal organs manifest through changes in the tongue. In TCM, the formation of tongue coating is attributed to the disharmony of *Zang-Fu* organs, with “fumigation of the spleen and stomach” leading to increased metabolism, heightened tongue blood flow, and thickening of the coating. Additionally, nutrient metabolism, particularly involving cofactors and vitamins, influences the nutritional status, metabolic activity, and *Zang-Fu* functions, indirectly affecting the tongue coating. In TCM, diabetes is classified under “collateral disease,” characterized by symptoms such as excessive thirst, frequent urination, and weight loss, which corresponds to the term “thirst-quenching” (Liu et al., 2024). Diabetes is primarily considered a result of “humidity” and “heat” imbalances in the body (Li et al., 2024a). Tongue coating in Pre-DM and T2DM patients often appears thick and greasy, with a rough texture and aged appearance. Color parameters indicate that both tongue and moss colors progressively become pale and white.

Research has increasingly established a clear association between gut microbiota dysregulation and the onset of T2DM (Sharma and Tripathi, 2019). Diabetic patients exhibit significant alterations in gut microbiota composition compared to healthy individuals, with microbial biodiversity in Pre-DM and T2DM progressively declining (Yang et al., 2021). This observation aligns with the current study’s findings, where both the Observed Species and Shannon index of intestinal flora in Pre-DM and T2DM patients were markedly lower than in healthy controls, indicating a reduction in microbial richness associated with diabetes. In metabolic disorders, the gut microbiome is frequently characterized by dysbiosis, typically manifesting as a reduction in commensal bacteria alongside an increase in pathogenic species. This shift results in an overrepresentation of normally minor bacterial populations, particularly opportunistic pathogens, and a corresponding decline in overall diversity (Scheithauer et al., 2020). Throughout the progression from pre-glucose intolerance to T2DM, bacterial changes often follow a synchronous pattern (Allin et al., 2018), a trend that this study also confirmed. Specifically, gut microbiota composition evolved with diabetes progression, evidenced by an increase in *Bacteroides-H* and a decrease in *Faecalibacterium* and Bifidobacterium. The most pronounced increase in *Phocaeicola* was observed in the Pre-DM group, while *Escherichia* was significantly enriched in T2DM, holding the highest relative abundance. These trends are consistent with the findings of Li Wang et al. (Wang et al., 2021) and Christian Diener et al (Diener et al., 2021). Additionally, Xiuying Zhang et al. (Zhang et al., 2013) demonstrated that the relative abundance of Bacteroides fluctuates significantly in response to worsening glucose intolerance.

In this study, the analysis integrated oral flora alongside intestinal flora, revealing a notable contrast between the two. The oral microbiota showed a marked increase in species abundance in both Pre-DM and T2DM groups compared to the gut microbiota. This discrepancy may be attributed to the thickened tongue coating observed in patients, in contrast to the thin coating in healthy individuals. As the disease progresses, the accumulation of phlegm and dampness leads to a thicker, greasier tongue coating, corresponding with an increase in oral microbial diversity. Further species composition analysis identified significant enrichment of *Pauljensenia* and *Veillonella-A* in the T2DM group, while Neisseria exhibited a slight decrease, consistent with the trends observed in Pre-DM. Additionally, *Haemophilus-D* and *Porphyromonas-A* were reduced exclusively in the T2DM group. LEfSe analysis indicated that *Porphyromonas* may serve as a key marker distinguishing Pre-DM from T2DM, given its association with chronic inflammatory diseases. Insulin resistance, thus, seems secondary to the inflammatory process, where both innate and adaptive immune responses are potentially driven by microbiota-induced inflammation (Cai et al., 2011; Agrawal and Kant, 2014). These results highlight the pivotal role of inflammation in the development and progression of diabetes.

To investigate the association between TCM tongue diagnosis and microbiota, patients with thin white fur and white greasy fur were selected to examine the correlation between tongue characteristics and the oral-gut microbiota axis. Analysis of species composition revealed that the predominant flora in both the oral cavity and intestines of patients with white greasy coating belonged to Firmicutes, a key producer of butyric acid among short-chain fatty acids (SCFAs). SCFAs play a role in fatty acid oxidation, glucose metabolism, and inflammation (Rivera-Chávez et al., 2016), with butyric acid promoting cytokine production in anti-inflammatory regulatory T cells and enhancing lipolysis (Atarashi et al., 2011, 2013; Rumberger et al., 2014). To further understand the mechanism behind greasy fur formation, an abundance analysis of KEGG functional pathways was performed. Results indicated that the differential changes in tongue coating were primarily linked to pathways involved in Metabolism, with the highest abundance observed in metabolic cofactors and vitamins.

Advancements in science and technology have accelerated the application of deep learning models in disease diagnosis and classification (Zhang et al., 2024a). RF analysis and ANN models have been employed to identify key signature genes and develop diagnostic frameworks (Yao et al., 2024). Xiaozhou Lu et al. (Lu et al., 2024) demonstrated that deep learning-based tongue image analysis serves as an effective screening tool for liver fibrosis. In previous research, tongue image analysis was integrated with microbiome technology to develop an early screening model for MAFLD with enhanced accuracy (Dai et al., 2024). This study marks the first instance of combining tongue analysis and microbiome data for the prediction and diagnosis of Pre-DM and T2DM, achieving high precision. Among the three diagnostic models, SVM showed the highest accuracy, reaching 78.9%. Contribution analysis identified TB-a and perALL in tongue image parameters, along with *Escherichia* and *Porphyromonas-A* in microbiota, as primary classification indicators for Pre-DM and T2DM diagnosis. Non-laboratory-based risk models offer the potential to identify and prioritize individuals at higher risk, guiding targeted diagnostic testing and preventive measures. Constructing a diagnostic model confirms the importance of tongue image and oral flora in diagnosing diabetes, providing a basis for further exploration of the mechanism.

This experiment led to several key conclusions. First, distinct characteristic markers are present at various stages of diabetes progression. The increased abundance of *Porphyromonas* in the oral microbiota and *Blautia* in the gut microbiota not only serve as microbiological indicators for Pre-DM patients but also function as risk predictors for T2DM. *Escherichia* is significantly elevated in T2DM and represents a potential microbial marker for the condition. Additionally, the study identified alterations in tongue imagery and the oral-gut axis across different stages of diabetes, emphasizing the central role of elevated *Firmicutes* in the oral-gut axis in the development of a white, greasy coating, closely linked to metabolic processes. A diagnostic and predictive model was also developed, achieving high accuracy through the SVM model. Parameters such as TB-a and perALL in tongue imagery, along with *Escherichia* and *Porphyromonas-A* in microbiota, emerged as primary classifiers for Pre-DM and T2DM diagnosis. The findings further underscore the relevance of the oral microbiota present on tongue coatings, supporting the scientific foundation of TCM tongue diagnosis in disease management.

This exploratory research combined tongue diagnosis from TCM with analysis of the oral-gut microbiome axis. However, limitations due to the small sample size led to overfitting in the machine learning model, and validation of the corresponding diagnostic model remains insufficient. Future studies will aim to address these issues by increasing the sample size, controlling for confounding variables, and incorporating additional bioinformatics data to further identify diagnostic markers for Pre-DM and T2DM.

## Data Availability

The datasets presented in this study can be found in online repositories. The names of the repository/repositories and accession number(s) can be found in the article/supplementary material.
